# Taxonomic evaluation of misidentification of crude herbal drugs marketed in Iran

**Published:** 2012

**Authors:** Mohammad Reza Joharchi, Mohammad Sadegh Amiri

**Affiliations:** 1 *Ferdowsi University of Mashhad, Mashhad, I. R. Iran*; 2 *Department of Biology, Payame Noor University, Tehran, I. R. Iran*

**Keywords:** Adulteration, Authentication, Crude Herbs, Identification, Iran, Market

## Abstract

**Objective: **Medicinal plants organize an effective source of folk and modern medicine. Correct identification, authentication and quality control are essential to ensure safety, therapeutic potency, efficacy and reproducible quality of herbal medicines. The aim of this study is to use taxonomic method for authentication of traditional herbal drugs which are commonly sold in herbal shops in Iran. In this regard, twenty-seven cases of herbal drugs suspected to be adulterated were investigated.

**Material and Methods:** Crude raw material of herbal drugs was prepared from the various markets in Iran and was identified at the Ferdowsi University of Mashhad Herbarium (FUMH).

**Results: **Taxonomic evaluation revealed that 78 species belonging to 21 families which are traded in Iranian market should be considered as authentic, adulterated and substituted samples.

**Conclusion: **It was concluded that nowadays, many of the medicinal plants available in the market have ambiguous identification along with adulteration and contamination. The present study provides awareness amongst the traders, researchers, clinicians and manufacturing units about the ambiguity of authenticity in the traded herbal raw materials.

## Introduction

Since ancient times, plants have been one of the first and most available resources usable for treating illnesses, and throughout history, there has always been a close relationship between man and plants, and the medicinal effects of plants and their uses have been known by everybody (UNESCO, 1996 ▶). Currently, according to the World Health Organization (WHO), as many as 80% of the world's people, depend on traditional medicine for their primary health care needs. There are considerable economic benefits in the development of indigenous medicines and in the use of medicinal plants for the treatment of various diseases (Azaizeh et al., 2003 ▶). Traditional medicine involves the use of plant parts in crude form, either fresh or dried for preventing or healing various forms of ailments. Due to some morphological similarities of the plant parts and their improper identification by the consumers and herbal plant sellers and lack of a standard identification system, the crude medicinal plants and their parts are often adulterated or substituted in commerce which may result in the loss of their efficacy and tendency to toxicity. Correct identification of herbal drug is the foundation of safe use of herbal medicines and products. Without proper identification as a starting point, the safe use of quality products cannot be guaranteed. There is recognition within industry and government that there is a need to protect access and selection by consumers when it comes to natural health products. At the same time, consumers have a right to expect that these products can be used with confidence regarding their safety and quality (Ahmad et al., 2009 ▶). Dried products sold in the market are generally difficult to identify, as many useful diagnostic characteristics are lost during drying. At the same time, other numerous problems are confronted to taxonomists in the identification of traded herbal drugs. The existence of several common names for the same plant species in different areas may confuse end users for selection and utilization of a genuine drug. Another problem is superficial resemblance of plant species within the same tribe or family (Khan et al., 1996 ▶). Problem of adulteration in medicinal plants arose due to the potential use of different species for similar ailments (Shinwari et al., 2002 ▶). Iran has a very honorable past in traditional medicine, which goes back to the time of Babylonian-Assyrian civilization. One of the most significant ancient heritages is sophisticated experience of people who have tried over the millennia to discover useful plants for health improvement and each generation adding their own experience to this tradition (Naghibi et al., 2005 ▶). 

Nowadays, on the one hand lack of quality control, adultration, substitution, and improper storage are known to decrease the efficacy of traditional medicines and on the other hand there exists a lot of confusion regarding the botanical identity of many medicinal drugs in Iran. In this study, taxonomic evaluation of medicinal plant species and market samples were carried out to find out what is the nature of the trade samples.

## Material and Methods

During 2009-2010, an attempt was made for authentication of traditional herbal drugs which are commonly traded in herbal shops in Iran. The raw material of herbal drugs were procured from the various markets in Iran i.e. Esfahan and Mashhad bazaars, and Shiraz, Tabriz, and Tehran herbal shops. Among the many cases discovered, twenty-seven cases were selected. The plant samples were later examined thoroughly at the Ferdowsi University of Mashhad Herbarium (FUMH) for proper identification. In this regard, the samples were cleansed from foreign matter and their parts were examined for morphological characteristics after placing underusing a dissecting microscope. Subsequently, correct identification was made with the help of the various Floras (Rechinger, 1963-2005 ▶; Assadi et al., 1988-2008 ▶) and consulting with different herbal literature (Amin, 1991 ▶; Hooper, 1937 ▶; Zargari, 1989-1992 ▶). The voucher specimens were later deposited in the FUMH for further reference. 

## Results

Table 1 illustrates the results of this work.

**Table 1:** Results of taxonomic evaluation of misidentification of crude herbal drugs marketed in Iran. Column 1: Authentic sample, Column 2: Adulterated or substituted sample.

**No.**	**Drug name & Part used**	**Taxa Identified**	**1**	**2**	**Family**	**Remarks**
1	**Afsantin** **(Flower)**	*Artemisia absinthium *L*.*	*		Asteraceae	Indigenous
2		*Helichrysum graveolens* Sweet		*	Asteraceae	Indigenous
3	**Aftimoon** **Aerial parts)** **)**	*Cuscuta* *epithymum* Murray	*		Convolvulaceae	Indigenous
4		*Cuscuta* *australis* R.Br.		*	Convolvulaceae	Indigenous
5		*Cuscuta* *planiflora* Ten.		*	Convolvulaceae	Indigenous
6	**Alaf-e- simkesh** **Aerial parts)** **)**	*Trichodesma* *incanum* Bunge	*		Boraginaceae	Indigenous
7		*Sophora* *pachycarpa* Schrenk ex C.A.Mey.		*	Fabaceae	Indigenous
8	**Anjedan-e-romi** **(Fruit)**	*Levisticum* *officinale* W.D.J.Koch	*		Apiaceae	Indigenous
9		*Zosima* *absinthifolia* Link		*	Apiaceae	Indigenous
10	**Badranjbuyeh** **Aerial parts)** **)**	*Melissa* *officinalis* L.	*		Lamiaceae	Indigenous
11		*Hymenocrater* *elegans* Bunge		*	Lamiaceae	Indigenous
12		*Hymenocrater* *bituminosus* Fisch. & C.A.Mey.		*	Lamiaceae	Indigenous
13		*Hymenocrater* *calycinus* Benth.		*	Lamiaceae	Indigenous
14		*Hymenocrater* *platystegius* Rech.f.		*	Lamiaceae	Indigenous
15		*Dracocephalum* *moldavica* L.		*	Lamiaceae	Indigenous
16		*Asperugo* *procumbens* L.		*	Boraginaceae	Indigenous
17	**Balangu-e- shirazi** **(Seed)**	*Lallemantia* *iberica* Fisch. & C.A.Mey.	*		Lamiaceae	Indigenous
18		*Lallemantia* *royleana* Benth.	*		Lamiaceae	Indigenous
19		*Ocimum* *basilicum* L.		*	Lamiaceae	Indigenous
20		*Dracocephalum* *moldavica* L.		*	Lamiaceae	Indigenous

Column 1: Authentic sample, Column 2: Adulterated or substituted sample.

## Discussions

Taxonomy is a human invention which intends to create a system of classification that can be used by all who are concerned about the differences and similarities among organisms (Cronquist, 1988 ▶). Taxonomic evaluation of the drug is one of the most important steps toward quality maintenance. Medicinal plants are generally collected by professionals who may not be botanists or taxonomists. Similarly, the identity of crude drugs purchased from the market which is based on trade or vernacular name is taken for granted, without subjecting the plant material for stringent method of botanical identification (Ahmed et al., 2005 ▶).

In addition to nomenclatural ambiguity, the traditional drugs sold in herbal shops in Iran are adulterated or substituted with quite unrelated plant materials. Herbal adulteration is one of the common malpractices in herbal raw material trade. Adulteration is described as intentional substitution of the original plant with another plant species or intentional addition of a foreign substance to increase the weight or potency of the product or to decrease its cost. In general, adulteration is considered as an intentional practice. With our experience, it is noted that the herbal drugs are also adulterated unintentionally. Unintentional adulteration may be due to the following reasons:

1. Confusion in vernacular names between indigenous systems of medicine and local dialects

2. Lack of knowledge about the authentic plant

3. Unavailability of the authentic plant

4. Similarity in morphology or aroma

5. Careless collection

According to Table 1, real babooneh (*Matricaria recutita*) with 6 other species belonging to family Asteraceae (i.e. *Anthemis nobilis*,* Anthemis wiedemanniana, Tripleurospermum disciforme*, *Tanacetum parthenium*,* Tanacetum persicum,* and *Microcephala lamellata*) and also real Badranjbuyeh (*Melissa officinalis*) with 6 other species (i.e.* Hymenocrater elegans*,* Hymenocrater bituminosus*, *Hymenocrater calycinus*,* Hymenocrater platystegius*, *Dracocephalum moldavica,* and* Asperugo procumbens*) were found to be the most adulterated or substituted in the market samples in Iran. Some species mentioned in table 1 do not grow naturally in Iran and could have been cultivated or imported from other countries (i.e.* Borago officinalis*, *Lavandula angustifolia,* and *Valeriana officinalis*). There are only two species of *Lavandula *growing naturally in Iran,* L. stricta *Del. and *L. sublepidota *Rech. f. These species are not mentioned as medicinal plants in the references. Citation of species which do not occur in Iran may also be a result of misidentification of these plants.

The importance of a correct scientific identification of plants can hardly be exaggerated, since it is the only key connecting the ethnobotanical information gained with already existing biological and chemical knowledge recorded in the literature. However, vernacular synonyms in the literature search pose a major problem. In some of the traditional texts, it is not possible to match these names with scientific names. 

Another problem is the uncertainty regarding scientific naming of plants, because of the different vernacular names or a local name which is given to two or more species. For example, the name Zoofa is matched with *Hyssopus officinalis *L. and three species of *Nepeta *(i.e. *Nepeta bracteata*, *Nepeta ispahanica,* and* Nepeta micrantha*) in different references. Badranjbuyeh has been variously referred to the species of *Hymenocrater*, *Dracocephalum,* *Asperugo, *and *Melissa *or the name Afsantin is coupled with two species of *Helichrysum graveolens* Boiss. and* Artemisia absinthium* L. in different parts of Iran. Local names are not reliable for identification of plants, because they differ significantly from one region to another. Marketing under common names has added to the confusion, since different plants may have the same common name in different parts of the world such as a drug named Gavzaban with two authentic samples (i.e. *Borago officinalis* and* Echium amoenum*). *Borago officinalis* L. is not indigenous to Iran and is relevant to semi-arid regions of western Europe whereas *Echium amoenum* Fisch. & Mey. grows widly in the northern highlands of Iran. *Alcea *spp. with (40 sp*.*) especially (*Alcea rosea*,* Alcea aucheri*,* Alcea angulata,* *Alcea rhyticarpa,* and *Alcea lavateriflora*) are known as Gol-e-khatmi in different parts of Iran and in some market samples, *Hibiscus syriacus* L. adulterated or substituted instead of them. Distinction and identification of drug are very important because the adulterants, although belonging to the same genus as the drug, does not possess the medicinal properties of the drug. For instance,* Bunium cylindricum* are mixed with real Zire-e-siah (*Bunium persicum*) and are sold in the market resulting in the degrading of the quality and efficacy of the drug. 

It was concluded that nowadays many of the medicinal plants available in the trade have ambiguous identification along with adulteration and contamination. Moreover, labels on herbal products do not mention correct plant species, due to the lack of services of taxonomic or botanical expertise. Therefore, in order to ensure safety, therapeutic potency, and efficacy of herbal medicines, correct identification, authentication, and elimination of adulteration are essential. After completing these formalities, the drug should only be authenticated by a panel of experts including taxonomists. Furthermore, suppliers and traders should be educated about the authentic sources. Suppliers are illiterate and not aware about their spurious supply. Even scientific community and traditional physicians are unaware of it. It is absolutely vital that organizations such as Iran Medical Research Council support and promote regional certification facilities to set gold standards for medicinal plants. This survey thus warns all traders, practitioners, traditional physicians, manufacturing units, and scientific community for an urgent need for the selection of authentic raw materials in the herbal market.
